# Dsg2 Upregulation as a Rescue Mechanism in Pemphigus

**DOI:** 10.3389/fimmu.2020.581370

**Published:** 2020-10-28

**Authors:** Anna M. Sigmund, Letyfee S. Steinert, Desalegn T. Egu, Franziska C. Bayerbach, Jens Waschke, Franziska Vielmuth

**Affiliations:** Department I, Faculty of Medicine, Institute of Anatomy and Cell Biology, Ludwig-Maximilians-Universität, Munich, Germany

**Keywords:** pemphigus, autoimmune blistering diseases, epidermis, keratinocyte, desmosome

## Abstract

In pemphigus vulgaris (PV), autoantibodies directed against the desmosomal cadherin desmoglein (Dsg) 3 cause loss of intercellular adhesion. It is known that Dsg3 interactions are directly inhibited by autoantibody binding and that Dsg2 is upregulated in epidermis of PV patients. Here, we investigated whether heterophilic Dsg2-Dsg3 interactions occur and would modulate PV pathogenesis. Dsg2 was upregulated in PV patients’ biopsies and in a human *ex vivo* pemphigus skin model. Immunoprecipitation and cell-free atomic force microscopy (AFM) experiments demonstrated heterophilic Dsg2-Dsg3 interactions. Similarly, in Dsg3-deficient keratinocytes with severely disturbed intercellular adhesion Dsg2 was upregulated in the desmosome containing fraction. AFM revealed that Dsg2-Dsg3 heterophilic interactions showed binding frequency, strength, Ca^2+^-dependency and catch-bond behavior comparable to homophilic Dsg3-Dsg3 or homophilic Dsg2-Dsg2 interactions. However, heterophilic Dsg2-Dsg3 interactions had a longer lifetime compared to homophilic Dsg2-Dsg2 interactions and PV autoantibody-induced direct inhibition was significantly less pronounced for heterophilic Dsg2-Dsg3 interactions compared to homophilic Dsg3 interactions. In contrast, a monoclonal anti-Dsg2 inhibitory antibody reduced heterophilic Dsg2-Dsg3 and homophilic Dsg2-Dsg2 binding to the same degree and further impaired intercellular adhesion in Dsg3-deficient keratinocytes. Taken together, the data demonstrate that Dsg2 undergoes heterophilic interactions with Dsg3, which may attenuate autoantibody-induced loss of keratinocyte adhesion in pemphigus.

## Introduction

Desmosomes are highly regulated protein complexes mediating strong intercellular adhesion in tissues constantly exposed to high mechanical stress such as skin and heart (1, 2). They are composed of the desmosomal cadherins desmoglein (Dsg) 1-4 and desmocollin (Dsc) 1-3, which mediate intercellular adhesion in a Ca^2+^-dependent manner (3). Desmosomal cadherins are linked *via* plaque proteins such as plakoglobin (PG), plakophilins (Pkp), and desmoplakin (DP) to the intermediate filament cytoskeleton (4).

Desmosomal cadherins are known to interact with their counterparts in a homo- and heterophilic manner (5–8). For those interactions, an interaction mechanism comparable to the tryptophan swap model of classical cadherins is proposed. According to this model, a conserved tryptophan at position 2 binds in a hydrophobic pocket at the EC1 of the binding partner on the opposing cell membrane (trans-) or on the same cell (cis-interaction) (3). In the epidermis, desmosomal proteins show a differentiation and thus layer-specific expression pattern (9). For instance, Dsg1 and Dsg3 show a reversed distribution gradient with predominant expression of Dsg3 in the basal layers.

The importance of desmosomal proteins for tissue integrity is reflected in several desmosome-related diseases such as the genetic disorder arrhythmogenic cardiomyopathy or the bullous autoimmune disease pemphigus (10). The autoantibody profile of the latter correlates with the clinical phenotype of the disease (11). In pemphigus vulgaris (PV), autoantibodies mainly directed against Dsg1 and 3 cause suprabasal blistering of the epidermis and mucous membranes, whereas in pemphigus foliaceus (PF) autoantibodies directed against Dsg1 cause superficial blisters in the skin only. Accordingly, Dsg3 knock out (ko) mice show a mild PV like phenotype with mainly oral lesions and PV typical loss of cell-cell adhesion in suprabasal layers (12, 13). In contrast, Dsg1 deficiency resulted in lethal PF-like superficial skin blistering in mice (14).

According to our current knowledge, pemphigus autoantibodies directly inhibit Dsg3 interactions (5, 8) and induce dysregulation of several signaling pathways (15). Activation of p38 mitogen-activated protein (MAP) kinase (16–18), sarcoma associated (Src) kinase (19, 20), extracellular signal-regulated kinase (ERK) (21, 22), and protein kinase C (23–25) by autoantibodies contribute to Dsg1 and 3 internalization and uncoupling of keratins from the desmosome subsequently leading to loss of intercellular adhesion and blister formation in PV. Besides Dsg1 and 3, numerous other non-desmoglein autoantibodies found in PV targeting important proteins in physiology and cell adhesion, which may contribute to pemphigus pathogenesis (26). Nevertheless, the exact pathomechanisms in pemphigus are not fully elucidated.

It was reported that Dsg2 is upregulated in PV patients’ lesions (27) and that Dsg2 and Dsg3 expression are interdependent in oral squamous cell carcinomas (28). Similarly, Dsg3-deficient keratinocytes showed a higher membrane localization of Dsg2 compared to wild type (wt) cells, and disturbed intercellular adhesion in Dsg3-depleted keratinocytes was impaired further after additional knock down of Dsg2 (29). Nevertheless, the biologic significance of Dsg2 upregulation for PV pathogenesis is unknown.

It has been shown that both Dsg2 and Dsg3 can undergo heterophilic binding with Dsc isoforms (8, 30). Further, Dsg3 binding events and unbinding forces were increased in keratin-deficient keratinocytes as shown by cell-free atomic force microscopy (AFM) measurements (6), and Dsg3 binding was partially blocked by a monoclonal inhibitory anti-Dsg2 antibody. Based on these observations, we speculated that in situations of compromised Dsg3-mediated cell adhesion, e.g., keratin-deficiency or pemphigus, Dsg2 may be capable of compensating for loss of intercellular adhesion. Therefore, we here examined the biophysical properties of heterophilic Dsg2-Dsg3 interactions and whether upregulation of Dsg2 affect pemphigus pathogenesis.

## Materials and Methods

For extended methods, please refer to the **Supplementary Materials and Methods**.

### Cell Culture and Establishment of Spontaneously Immortalized Mouse Keratinocytes

The stable mouse epidermal keratinocytes (MEK) cell line was generated by spontaneously immortalization from Dsg3 ko and wt B6;129X1-*Dsg3^tm1Stan^*/J mice as described before (Jackson Laboratory, Maine, US) (31). Briefly, epidermis was obtained from neonatal mice by incubation of the skin with Dispase II (Sigma-Aldrich, Munich, Germany) overnight. Epidermal cells were isolated after treatment for 1 h with Accutase (Sigma-Aldrich, Munich, Germany) and seeded in complete FAD media (0.05mM CaCl_2_, PAN Biotech, Aidenbach, Germany) on collagen I (rat tail; BD Bioscience, New Jersey, US) coated flasks and maintained at 35°C and 5% CO_2_. After passaging for 10–15 times, keratinocytes immortalized spontaneously, while other epidermal cells die. For experiments, confluent cells were switched to high Ca^2+^ (1.2 mM) for 48 h to induce differentiation.

Human keratinocyte cells (HaCaT) (32) were maintained in Dulbecco´s modified Eagle´s medium (DMEM) (Life Technologies; Carlsbad; CA; USA) supplemented with 10% fetal calf serum (Biochrom, Berlin, Germany), 50 units/ml penicillin (AppliChem, Darmstadt, Germany), 50 µg/ml streptomycin (AppliChem), and 1.8 mM Ca^2+^ at 37°C and 5% CO_2_.

### Purification of Recombinant Dsg-Fc Constructs

Purification of recombinant Dsg-Fc proteins was performed as described before (5). Briefly, extracellular domain-Fc constructs were expressed stably in Chinese hamster ovary (CHO) cells, and recombinant proteins were isolated from supernatants by protein A agarose affinity chromatography (Life Technologies/Thermo Fisher Scientific, Waltham, USA).

### Atomic Force Microscopy

Atomic force microscopy (AFM) utilizes a sharp tip on a flexible cantilever, which repetitively approaches and retrieves a probe. A laser beam is directed to the cantilever and its deflection, while the cantilever contacts the surface, is registered on a photodiode. During each approach-retract cycle, a force-distance curve is created by recording the deflection of the scanning tip. Analysis of force-distance curves allows to draw conclusions about the mechanical properties of the probe. By functionalizing the scanning tip and the probe with recombinant adhesion molecules, such as the extracellular domain of desmosomal cadherins, their respective interaction can be probed and the biophysical properties can be characterized (33, 34).

For AFM measurements, a NanoWizard 3 AFM (JPK Instruments, Berlin, Germany) mounted on an inverted optical microscope (Carl Zeiss, Jena, Germany) or a Nanowizard 4 AFM (JPK Instruments, Berlin, Germany) mounted on an inverted optical microscope (IX73 Olympus, Hamburg, Germany) were used in a cell-free setup described in detail before (6). Briefly, cantilever of silicon nitride MLCT AFM probes (Bruker, Mannheim, Germany) and silicon nitride mica sheets (SPI Supplies, West Chester, USA) were functionalized with corresponding Dsg-Fc constructs as illustrated before (35). A flexible heterobifunctional benzaldehyde polyethylenglycol (PEG) linker (BroadPharm, San Diego, US) was used to link proteins (0.15 mg/ml) to the respective surface. Measurements were done with the triangular D-tip with nominal spring constant of 0.03 N/m and tip radius of 20 nm using Force Mapping mode. By evaluation of the resulting force-distance curves, adhesive properties of specific molecules can be analyzed (36). Beside the probability of an interaction to occur, referred to as binding frequency, the strength of the interaction, so called unbinding force, can be determined.

### Keratinocyte Dissociation Assay

Dispase-based keratinocyte dissociation assay was performed as described before (37). Briefly confluent cells were switched to high Ca^2+^ for 48 h, and monolayer was detached from culture well by Dispase II (Sigma Aldrich) supplemented with 1% collagenase I (Thermo Fisher Scientific). Afterwards, a defined shear stress was applied, and resulting fragments were counted. Number of fragments reversely correlates with intercellular adhesion.

### Immunoprecipitation

Co-immunoprecipitation of HaCaT lysates (RIPA buffer, 50 mM Tris-HCl, pH 8; 150 mM NaCl; 0.1% SDS; 1% NP-40; 1 mM EDTA) using 1.5 µg of aDsg3 pAb (Biozol, Eching, Germany), aDsg2 pAb (Abbexa, Cambridge, UK) or a polyclonal rabbit ctr-IgG was performed as described previously (29). Briefly, 1 mg of precleared lysates were incubated together with the respective antibody for 3 h at 4°C on a rotating incubator. To precipitate antibody bound proteins, 30 µl of A/G beads (Santa Cruz Biotechnology, Dallas, TX, USA) were added for an overnight incubation at 4°C and subsequent centrifugation. Beads were washed with RIPA buffer, and precipitated proteins were analyzed by Western blotting. For co-immunoprecipitation after crosslinking of surface proteins, cells were treated with the membrane impermeable crosslinker DTSSP (3,3′-dithiobis[sulfosuccinimidylpropionate], ThermoScientific, Waltham, USA) according to the manufacturer´s instructions.

### Western Blot

Cell lysis and triton extraction were performed as described elsewhere in detail (37) and in **Supplementary Materials and Methods**. For detection of cell surface proteins, a biotinylation assay was performed using a protocol modified from Vielmuth et al. (38). In brief, confluent cells were incubated with membrane impermeable EZ-Link Sulfo-NHS-Biotin (ThermoScientific, Waltham, USA) and lysed in RIPA buffer (50 mM Tris-HCl, pH 8; 150 mM NaCl; 0.1% SDS; 1% NP-40; 1 mM EDTA) and centrifuged. Biotinylated surface proteins from pellet and supernatant were precipitated with NeutrAvidin HighCapacity agarose beads (Thermo Scientific, Waltham, USA) and analyzed by Western blot.

SDS-PAGE (SDS-polyacrylamide gel electrophoresis) followed by Western blot analyses was conducted as shown previously (37).

### *Ex Vivo* Pemphigus Skin Model

Human *ex vivo* pemphigus model was performed with cadavers of the human body donor program without history of skin diseases from the Institute of Anatomy and Cell Biology, Ludwig-Maximilians-Universität Munich, Germany as described before (17). Briefly, skin biopsis were divided into 1x1 cm pieces and intradermal injection of PV-IgG or ctr-IgG were conducted. Specimen were incubated for 24 h in DMEM at 37°C at 5% CO_2_ and subsequently subjected to a defined mechanical stress (six times scraping with constant pressure using a soft rubber). Finally, skin pieces were embedded in TissueTec (Leica Biosystems, Nussloch, Germany) for cryo cutting using a cryostat microtome (HM 500 OM, Microm International GmbH, Walldorf, Germany).

### Immunostaining

Staining of *ex vivo* and patient samples was performed after standard protocol as described before (17). Images were taken using a Leica SP5 confocal microscope with a 63x NA 1.4 PL APO objective controlled by LAS AF software (Leica, Mannheim, Germany).

### Ethic Statement

The studies involving human participants were reviewed and approved by Ethics Committee of the University of Würzburg (Az159/06), the Ludwig-Maximillians-Universität of Munich, the University of Budapest (Az48825-5/2019/EÜIG), the University of Marburg (Az20/14), and the University of Lübeck (Az12-178). The patients/participants provided their written informed consent to participate in this study.

### Data Processing and Statistics

Analysis of AFM force-distance curves was done with JPK data processing software and peak fit analysis and Bells equation was done in Origin Pro 2016, 93G (Northampton, MA). For processing and creation of figures, Adobe Photoshop CS4 (Adobe, Dublin, Ireland) was used. For graphs and statistics Prism 5 (GraphPad Software, San Diego, US) was used. For quantification of immunostainings, ImageJ-Software was used to determine the mean of raw intensities of epidermal cells divided by the area.

## Results

### Pemphigus Autoantibodies Induce Upregulation of Dsg2 in Human Epidermis

**Figure 1:**
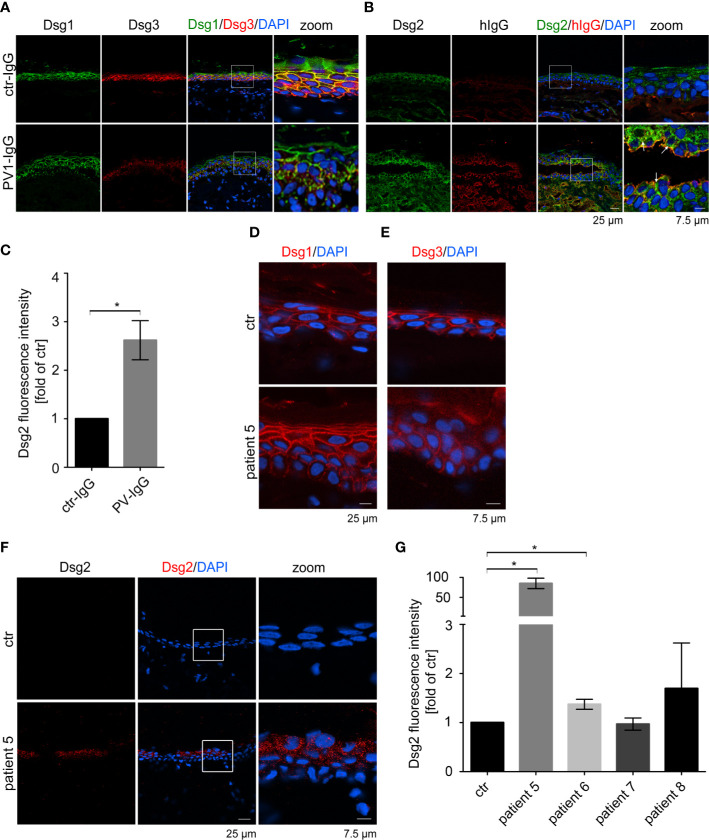
Pemphigus autoantibodies induce Dsg2 upregulation. Immunostaining against Dsg1, Dsg3 **(A)** and Dsg2 and human (h) IgG **(B)** in human *ex vivo* skin after injection with PV1-IgG or control (ctr) IgG from healthy humans. Dsg2 is upregulated in PV-IgG treated samples, especially along blisters (arrows). **(C)** Quantification of Dsg2 staining of perilesional cells of PV-IgG injected samples or control epidermal cells (n = 3 with PV1- and PV2-IgG). Pemphigus patient samples were stained against Dsg1 **(D)**, Dsg3 **(E)** and Dsg2 **(F)**. Untreated e*x vivo s*kin served as a control. Images show 1 of 2 Dsg2 positive samples out of 4 patients (patient 5-8). **(G)** Quantification of Dsg2 staining of patient 5–8 revealing upregulation of Dsg2 in pemphigus patients. Nuclei were stained with DAPI. Columns indicate mean value normalized to ctr ± SEM, *P < 0.05; student t-test to ctr. desmoglein (Dsg), pemphigus vulgaris (PV).

Dsg3 ko mice suffer from pemphigus-like lesions (12, 13). Primary keratinocytes derived from these mice show a highly disturbed intercellular adhesion. Interestingly, Dsg2, which is almost absent in healthy, adult epidermis, was localized along cell borders in these Dsg3 ko keratinocytes (29, 37). Thus, we wondered whether Dsg2 upregulation could serve as a compensatory mechanism in pemphigus. To investigate whether Dsg2 upregulation is present in pemphigus lesions, we used a human *ex vivo* pemphigus skin model. Healthy human skin from body donors was injected with either control IgG or PV1-IgG of pemphigus patient 1 (**Table 1**), incubated for 24 h, and subjected to mechanical stress. Samples injected with control IgG revealed normal expression patterns of Dsg1 and 3 with higher Dsg1 expression in the superficial epidermal layers and Dsg3 expression predominantly in the basal and suprabasal layers (**Figure 1A**). Dsg2 was almost absent along cell membranes as described before but showed diffuse cytoplasmic staining (**Figure 1B**) (37, 39). In contrast, PV1-IgG injection resulted in reduced and fragmented Dsg1 and Dsg3 staining, further referred to as Dsg depletion as described before (40) (**Figure 1A**). In contrast, Dsg2 staining in PV1-IgG injected samples was increased, which was particularly pronounced in keratinocytes surrounding the blisters where autoantibodies were bound predominantly (**Figures 1B**, arrows, **C**). Staining against human IgG confirmed binding of autoantibodies in PV-IgG injected samples only. Specificity of staining was shown by secondary antibody controls (**Figure S1A**). Similar results were obtained with injection of PV2-IgG (data not shown). To address specificity of this upregulation, we stained *ex vivo* samples for the desmosomal proteins Dsc1 and Dsc3. Both of them are described to be targets of autoantibodies in IgA pemphigus or PV, respectively (11, 41). Nevertheless, upregulation upon PV-IgG injection seemed to be specific for Dsg2 because neither Dsc1 nor Dsc3 staining in PV4-IgG injected samples was enhanced (**Figures S1B, C**). Similar results were obtained with injection of PV1- and PV2-IgG (data not shown).

**Table 1:** Dsg titers of pemphigus patient samples.

Patient	Dsg1 [U/ml]	Dsg3 [U/ml]	Clinical phenotype	Sample
1	168,1	174,64	PV	IgG
2	760	4711	PV	IgG
3	1,5	154	PV	IgG
4	n.d.	n.d.	PV	IgG
5	22	310	PV	skin
6	–	243	PV	skin
7	19,7	725	PV	skin
8	34	3038	PV	skin

n.d., Not defined; purified IgG samples of pemphigus patients (IgG), perilesional skin biopsies of pemphigus patients (skin). Cut-offs were at 20 U/ml.

Next, we analyzed Dsg2 expression in perilesional pemphigus patient samples (**Table 1**). Dsg1 staining of patient 5 was fragmented in the basal layers but not significantly reduced (**Figure 1D** and **Figure S2A**), but the patient showed Dsg3 depletion depicted by deffuse cytoplasmic staining (**Figure 1E** and **Figure S2B**). Interestingly, 2 out of 4 patient samples showed increased localization of Dsg2 at the membrane of epidermal keratinocytes, which was absent in all samples of healthy epidermis (**Figures 1F, G**). Binding of autoantibodies was tested by staining against human IgG and secondary antibody controls showed specificity of the staining (**Figures S2C, D**). Taken together, these data suggest that pemphigus autoantibodies induce Dsg2 upregulation.

### Dsg2 and Dsg3 Interact Heterophilically

*Ex vivo* and patient data suggest that Dsg2 could contribute to pemphigus pathogenesis as a compensatory mechanism. Former studies showed that Dsg3 interacts homo- and heterophilically with some desmosomal cadherins (5, 6, 8, 42). Thus, we here characterized whether Dsg3 would also undergo heterophilic interaction with Dsg2.

**Figure 2:**
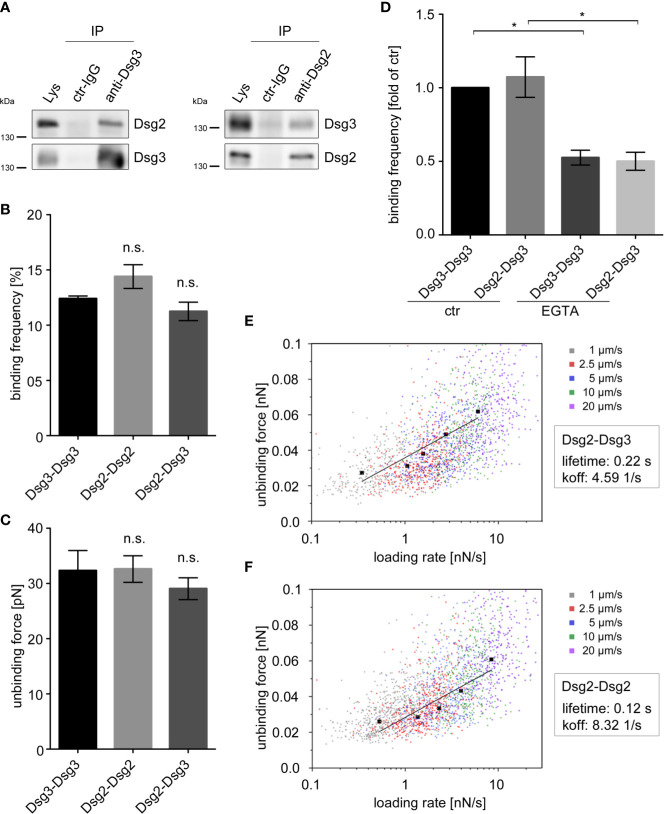
Dsg2 and Dsg3 interact heterophilically. **(A)** IP in human keratinocytes (HaCaT) with polyclonal anti-Dsg3 and anti-Dsg2 antibodies, or rabbit control (ctr) IgG showing heterophilic interactions of Dsg2 and Dsg3. Pure lysates (Lys) were loaded as a control. Representative of n = 4. Binding frequencies **(B)** and unbinding forces **(C)** of cell-free AFM measurements probing homophilic Dsg3-Dsg3, Dsg2-Dsg2 and heterophilic Dsg2-Dsg3 interactions. Binding frequencies and unbinding forces are comparable throughout all interaction types. Columns indicate mean value ± SEM (n = 5, each 2,500 force-distance curves). **(D)** Binding frequencies of cell-free AFM measurements probing Dsg3-Dsg3 and Dsg2-Dsg3 interactions with and without incubation of 5 mM EGTA for 1 h. Columns indicate mean value normalized to ctr ± SEM, *P < 0.05; One-way ANOVA with Bonferroni correction (n = 3, each 2,500 force-distance curves). Dot plots for Dsg2-Dsg3 **(E)** and Dsg2-Dsg2 **(F)** interactions of unbinding forces (UF) against logarithmic loading rates at various pulling speeds. Black graphs depict the extreme fitted values of UFs plotted against loading rates and fitted in a modified bells equation. Modified bells equation resulted in a bond lifetime of 0.22 s for heterophilic Dsg2-Dsg3 and of 0.12 s for homophilic Dsg2-Dsg2 interactions (n ≥ 3, each 2,500 **(E)** or 3,600 **(F)** force-distance curves). EGTA, ethylene glycol tetraacetic acid.

To investigate the heterophilic interaction of Dsg2 and Dsg3 in keratinocytes, we first performed immunoprecipitation using an anti-Dsg3 or anti-Dsg2 antibody in human keratinocytes (HaCaT). Indeed, Dsg2 co-immunoprecipitated with Dsg3 and vice versa showing the occurrence of heterophilic Dsg2-Dsg3 interactions in human keratinocytes (**Figure 2A**). We further applied the membrane-impermeable crosslinker DTSSP to assure extracellular interactions of Dsg2 and Dsg3. Indeed, IP after crosslinking confirmed interaction of Dsg2 and Dsg3 (**Figure S3A**).

Next, we characterized heterophilic Dsg2-Dsg3 interactions using cell-free AFM measurements. AFM measurements using tips and mica sheets coated with recombinant Fc-constructs of Dsg2- and Dsg3- extracellular domains revealed similar binding frequency for Dsg3-Dsg3 and Dsg2-Dsg2 homo- and Dsg2-Dsg3 heterophilic interactions (**Figure 2B**). Similarly, the strength of single molecule interaction, further referred as unbinding force (UF) did not differ between homophilic Dsg2-Dsg2 and Dsg3-Dsg3 interactions and heterophilic Dsg2-Dsg3 interactions (**Figure 2C**).

Similar to classical cadherins, homophilic interactions of desmosomal cadherins require Ca^2+^ (5, 43–45). However, it remains unclear whether heterophilic Dsg2-Dsg3 interactions reveal a similar Ca^2+^ dependency like homophilic interactions of Dsg2 and Dsg3. Thus, we depleted Ca^2+^ by application of the Ca^2+^-chelator EGTA in cell-free AFM measurements. EGTA significantly lowered the binding frequency of homophilic Dsg3-Dsg3 and of the heterophilic Dsg2-Dsg3 interactions to a similar extent (**Figure 2D**) indicating that heterophilic interactions are also Ca^2+^-dependent.

To further characterize the biophysical properties of heterophilic Dsg2-Dsg3 interactions, we determined their bond lifetime. AFM measurements with different pulling speeds from 1 to 20 µm/s revealed increasing UF with increasing pulling speeds and loading rates as depicted in a dot plot of UF against logarithmic loading rates at various pulling speeds (**Figure 2E**). This indicates that Dsg2-Dsg3 interactions behave like catch-bonds, which is in line with former studies on Dsg interactions (6, 34). By plotting the extreme fitted values of UFs against loading rates and fitting them in a modified Bells equation, we determined a bond lifetime of 0.22 s for heterophilic interactions (46, 47). This was almost doubled compared to lifetimes of homophilic Dsg2 interactions which were 0.12 s (**Figure 2F**).

Taken together, these data show that heterophilic Dsg2-Dsg3 interactions behave similar to the homophilic Dsg3 interactions in terms of binding frequency, strength, and Ca^2+^-dependency. In contrast, increased bond lifetime of the heterophilic Dsg2-Dsg3 interactions may stabilize keratinocyte adhesion under conditions when Dsg3-mediated cell adhesion is disturbed by pemphigus autoantibodies.

### Murine Dsg3 ko Keratinocytes Show Dsg2 Upregulation

To further analyze this potential compensatory Dsg2 upregulation mechanism we generated a stable murine Dsg3 ko keratinocyte cell line (MEK) from neonatal mice.

**Figure 3:**
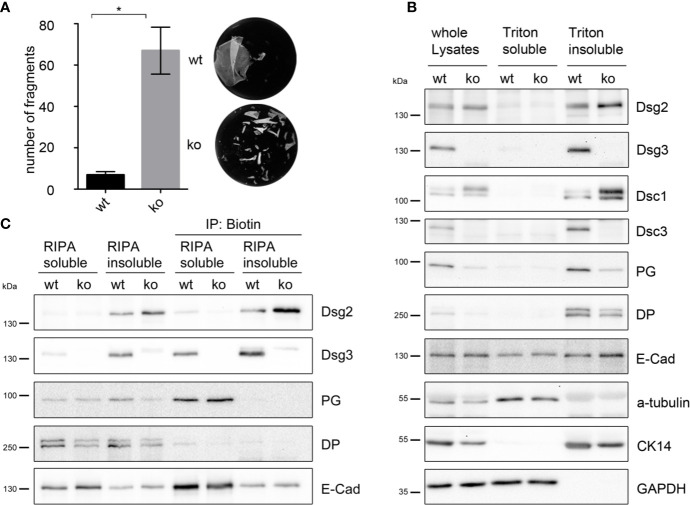
Dsg2 is upregulated in murine Dsg3 ko keratinocytes. **(A)** Dispase-based keratinocyte dissociation assay of stable Dsg3 wt and ko keratinocytes showing compromised intercellular adhesion in cells lacking Dsg3. Columns indicate mean value ± SEM, *P < 0.05; student t-test (n = 4). **(B)** Representative Western blot of Dsg3 wt and ko whole cell lysates, triton soluble and triton insoluble, desmosome containing fraction. Dsg3 ko cells show upregulation of Dsg2 and Dsc1 whereas Dsc3, cytokeratin 14 and the plaque proteins PG and DP were decreased compared to wt cells. E-Cad and GAPDH served as loading controls (n = 4). **(C)** Representative Western blot of a biotinylation assay. RIPA buffer soluble and insoluble biotinylated membrane proteins were immunoprecipitated using biotin coated beads demonstrating Dsg2 upregulation at the cell membrane of Dsg3-deficient keratinocytes. E-cad served as loading control (n = 4). DP, desmoplakin; CK, cytokeratin; Dsc, desmocollin; PG, plakoglobin; GAPDH, Glyceraldehyde 3-phosphate dehydrogenase.

Dispase-based keratinocyte dissociation assay showed a highly disturbed intercellular adhesion with 10 times more fragments in the Dsg3 ko MEKs compared to cells derived from wt littermates (**Figure 3A**).

Western blot analysis of whole cell lysates, triton soluble, and desmosome-containing triton insoluble fraction showed that expression of Dsg2 was also elevated in Dsg3 ko cells compared to wt cells, in line with the observation that pemphigus autoantibodies induce upregulation of Dsg2 in human epidermis (**Figure 3B**). Levels of E-Cad were unchanged in the corresponding fraction. Protein levels in the desmosomal fraction of Dsg3 ko lysates of Dsc3, cytokeratin 14, and the plaque proteins PG and DP were decreased, whereas Dsc1 levels were increased compared to wt cells (**Figure 3B**). Additionally cell surface biotinylation in Dsg3-deficient MEKs confirmed increased Dsg2 localization at the plasma membrane compared to wt cells (**Figure 3C**). Detection of E-Cad served as a loading control. Of note, Dsg2 seemed to be located in a fraction, which was in contrast to Dsg3, PG, and E-Cad not soluble in RIPA buffer. These results imply that lack of Dsg3 resulted in an upregulation of Dsg2 in desmosomes, which may contribute to rescue at least partly from disrupted cell cohesion.

### Heterophilic Dsg2-Dsg3 Interactions are Less Susceptible to Pemphigus Autoantibodies Than Homophilic Dsg3-Dsg3 Interactions

Pemphigus autoantibodies directed against Dsg3 cause direct inhibition of homophilic and heterophilic Dsg3 interactions (5, 8, 38). Thus, we wondered how pathogenic Dsg3 antibodies affect heterophilic Dsg2-Dsg3 interactions.

**Figure 4:**
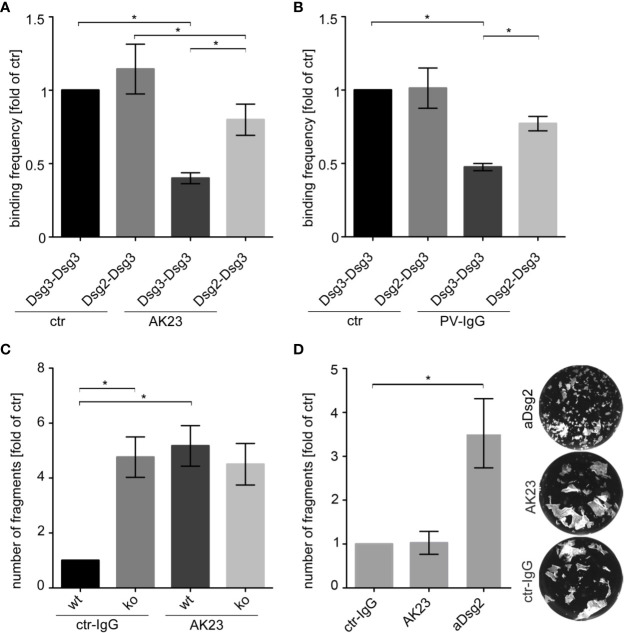
Heterophilic Dsg2-Dsg3 interactions were less susceptible to autoantibody-induced direct inhibition of Dsg interaction. Binding frequencies of cell-free AFM measurements probing Dsg3-Dsg3 and Dsg2-Dsg3 interaction pairs with and without (ctr) incubation of either a monoclonal anti-Dsg3 antibody (AK23) **(A)** or PV3-IgG **(B)** for 1 h (n ≥ 5, each 2500 force-distance curves). Heterophilic Dsg2-Dsg3 interactions are less susceptible against direct inhibition by pathogenic Dsg3 antibodies than homophilic Dsg3 interactions. **(C)** Dispase-based keratinocyte dissociation assay of Dsg3 wt and ko keratinocytes after treatment with control (ctr) IgG or AK23 for 24 h showing loss of intercellular adhesion after AK23 in wt cells only (n = 3). **(D)** Keratinocyte dissociation assay of Dsg3 ko keratinocytes after treatment with ctr-IgG, AK23 or an inhibitory anti-Dsg2 antibody (aDsg2) for 24 h revealing drastic effect of aDsg2 antibody in cells lacking Dsg3 (n = 4). Columns indicate mean value normalized to ctr ± SEM, *P < 0.05; One-way ANOVA with Bonferroni correction.

Therefore, we analyzed the heterophilic Dsg2-Dsg3 interactions by cell-free AFM measurements after treatment with AK23, a pathogenic anti-Dsg3 antibody, derived from a pemphigus mouse model (48). AK23 significantly reduced the binding frequency of homophilic Dsg3-Dsg3 interactions compared to untreated control (**Figure 4A**). In contrast, inhibition of heterophilic Dsg2-Dsg3 binding was less pronounced and AK23-treated heterophilic interactions showed a significant higher binding frequency compared to AK23-treated homophilic interactions. Moreover, AFM measurements also showed that Dsg2-Dsg3 interactions were less susceptible to PV3-IgG treatment than Dsg3-Dsg3 interactions (**Figure 4B**). In contrast to the pathogenic Dsg3-targeting autoantibodies, a monoclonal inhibitory anti-Dsg2 antibody inhibited both homophilic Dsg2-Dsg2 and heterophilic Dsg2-Dsg3 interactions to the same extent (**Figure S3B**).

Finally, we tested whether Dsg2 upregulation would protect to some extent against autoantibody-induced loss of cell cohesion. Therefore, we further analyzed the effect of AK23 on intercellular adhesion of murine keratinocytes in dissociation assays. AK23 increased the number of fragments in wt but not in Dsg3 ko MEKs (**Figure 4C** and **Figure S3C**). In contrast, treatment of Dsg3 ko cells with the inhibitory anti-Dsg2 antibody led to a drastic increase of fragments, implying that upregulated Dsg2 can partially compensate for deficiency of Dsg3 and thereby ameliorate loss of intracellular adhesion (**Figure 4D**).

## Discussion

Our data show an upregulation of Dsg2 in a human *ex vivo* pemphigus skin model and in pemphigus patient epidermis. Upregulation of Dsg2 allowed heterophilic interactions of Dsg2 with Dsg3. Because biophysical properties of heterophilic interactions of Dsg2-Dsg3 are in part different from homophilic Dsg interactions, the data indicate that heterophilic interactions may be advantageous. First, these heterophilic interactions show a higher bond lifetime than homophilic Dsg2-Dsg2 interactions. More important, they were less susceptible to direct inhibition by pathogenic pemphigus antibodies than homophilic Dsg3-Dsg3 interactions. Taken together, these data argue for a compensatory mechanism in pemphigus based on heterophilic Dsg2-Dsg3 interactions.

### Biological Impact of Homo- and Heterophilic Dsg3 Interactions

Desmosomal cadherins were reported to undergo homophilic and heterophilic interactions (8, 34, 42, 49). Data obtained by bead aggregation assays and surface plasmon resonance using the first extracellular domain of desmoglein isoforms indicated predominant occurrence of heterophilic interactions (8, 42). Nevertheless, in the study of Harrison et al., crystal structure analyses demonstrated homodimers of Dsg2 if high concentrations of complete molecules were applied which may reflect the situation in densely packed desmosomes (42). Using AFM, heterophilic interactions of Dsg2 with Dsc2 and Dsg2 with EGFR and Dsg1 with Dsc3 (7, 30, 50, 51), and homophilic interactions for all desmogleins were detected (6, 34). Biochemical cross-linking indicated that in keratinocytes homophilic interactions of Dsg isoforms are predominant (49). Collectively, these data indicate that Dsg can undergo both homophilic and heterophilic interactions. Possible differences may be caused by the techniques and model systems applied in which the mechanical load on the molecules differs. This was reported to be crucial for the function of several parts of cell-cell-adhesion structures (1, 52, 53). Further, distinct interactions could be preferred in dependency on the biological circumstances, differentiation of the cells and cellular behavior. Therefore, for this study, we combined biochemical methods and AFM analyses to study whether Dsg3 can undergo interactions with Dsg2. By co-immunoprecipitation of Dsg2 with Dsg3, we confirmed heterophilic interactions in human keratinocytes. However, IP data do not exclude the possibility of an interaction of Dsg2 and Dsg3 by cytoplasmic proteins. Thus, we further used cell-free AFM and detected both homophilic interactions of Dsg2 and Dsg3 and heterophilic interactions of Dsg2 with Dsg3, which is in accordance to previous studies (6, 38, 54). Taken together, the data presented strongly argue for a direct interaction of Dsg2 and Dsg3 *via* their extracellular domains.

Both interaction types, i.e. homo- and heterophilic, were Ca^2+^-dependent and behaved like catch-bonds, which was described to be characteristic for cadherin-type adhesion (34, 55, 56). Catch-bonds are characterized by binding strength which increases with the load applied on the interaction (57). Interestingly, forces and binding probability were comparable for all interactions detected, whereas bond lifetime for heterophilic interactions was prolonged in comparison to homophilic interactions of Dsg2. Similar results were reported for the homophilic interactions of Dsg1 in comparison to the heterophilic interactions of Dsg1 to Dsc3 (7). Thus, one can speculate that homo- and heterophilic interactions may fulfill different biological functions and heterophilic interactions may be beneficial under conditions where intercellular adhesion is challenged such as in pemphigus.

### Dsg2 Is Upregulated When Homophilic Interactions of Dsg3 are Disturbed and may Protect Against PV-IgG-Induced Loss of Cell Adhesion

Dsg2, except in hair follicles, is almost absent in healthy adult human epidermis (37, 36). In contrast, Dsg2 was reported to be upregulated in perilesional skin of pemphigus patients (27). Our data confirm upregulation of Dsg2 in pemphigus patient skin. Further, Dsg2 upregulation was present in a human *ex vivo* pemphigus model at membranes of cells bordering blisters suggesting that PV-IgG-induced loss of adhesion was causative for this phenomenon. This observation is in line with the hypothesis that perturbed Dsg3 adhesion caused upregulation of Dsg2 and both homophilic Dsg2-Dsg2 and heterophilic Dsg2-Dsg3 interactions may compensate for loss of Dsg3 function in pemphigus. This supports previous data in cultured murine keratinocytes lacking Dsg3 in which Dsg2 was reported to partly compensate for loss of intercellular adhesion (29, 37). Interestingly, comparable to our *ex vivo* model, immunostaining of Dsg3-deficient keratinocytes revealed an increase of Dsg2 at cell membranes (37). Here, we further generated a stable Dsg3-deficient cell line, which showed disturbed cell adhesion and upregulation of Dsg2 in the insoluble, desmosome-containing fraction supporting the observations revealed by immunostaining of *ex vivo* and pemphigus patient epidermis. Taken together, these data suggest that Dsg3 function and Dsg2 expression may be interdependent.

Keratinocytes are capable of inducing rescue mechanisms to protect against PV-IgG-induced loss of intercellular adhesion. For instance, a cAMP increase was reported after PV-IgG treatment in human keratinocytes—a mechanism that is effective to prevent loss of intercellular adhesion when augmented by cAMP increasing mediators (58). Similarly, Dsg2 upregulation could also serve as a cellular rescue mechanism in PV. This is in line with a study showing that mice after forced expression of Dsg2 in superficial epidermal layers were protected against PF-IgG-induced blister formation (59).

Therefore, it is conceivable that Dsg2 upregulation may also protect against loss of Dsg3 adhesion in response to antibody binding. It is known that Dsg3 interactions are disturbed by direct inhibition caused by autoantibodies (5, 8). Interestingly, heterophilic interactions between Dsg2 and Dsg3 were less susceptible to inhibition of trans-interaction in response to both the pathogenic Dsg3 antibody AK23 and PV-IgG. Importantly, a specific inhibitory anti-Dsg2 antibody further impaired loss of intercellular adhesion in Dsg3 ko keratinocytes, supporting the idea of a compensatory mechanism.

Taken together, the data support the hypothesis of an autoantibody-induced rescue mechanism based on Dsg2 upregulation and Dsg2-Dsg3 heterophilic interactions to ameliorate loss of intercellular adhesion in pemphigus.

## Data Availability Statement

The raw data supporting the conclusions of this article will be made available on request by the authors, without undue reservation.

## Ethics Statement

The studies involving human participants were reviewed and approved by Ethics Committee of the University of Würzburg (Az159/06), the Ludwig-Maximillians-Universität of Munich, the University of Budapest (Az48825-5/2019/EÜIG), the University of Marburg (Az20/14), University of Lübeck (Az12-178). The patients/participants provided their written informed consent to participate in this study. The animal study was reviewed and approved by the Government of Upper Bavaria, (Az. 55.2-2532.Vet_02-14-139).

## Author Contributions

AS and FV conducted the experiments, acquired the data, and analyzed the data. AS, FV, and DE developed the methodology. FB and LS conducted the experiments. AS, FV, and JW designed the research studies. AS, FV, and JW wrote the manuscript. All authors contributed to the article and approved the submitted version.

## Funding

This work was supported by DFG FOR 2497 TP5 to JW and TP6 to FV and Else-Kröner-Fresenius-Stiftung 2016_AW157 to FV and JW.

## Conflict of Interest

The authors declare that the research was conducted in the absence of any commercial or financial relationships that could be construed as a potential conflict of interest.
