# Supplementary material for: Dsg2 Upregulation as a Rescue Mechanism in Pemphigus
Source: Front Immunol. 2020 Oct 28;11:581370. doi: 10.3389/fimmu.2020.581370 (PMC7655986; doi:10.3389/fimmu.2020.581370)
Supplement: Supplementary file 1 [file DataSheet_1.pdf]

Supplemental material and methods

Antibodies

For Western blotting primary antibodies against Dsg3 polyclonal antibody (pAb) (Biozol, Eching, Germany), Dsg2 pAb (1:1000 dilution, Abbexa, Cambridge, UK), Dsc1 pAb (1:1000 dilution, Abcam, Cambridge, UK), Dsc3 pAb (1:1000 dilution, LS Bio,Seatle, US), plakoglobin monoclonal antibody (mAb) (PG 5.1, 1:1000 dilution, Progen, Heidelberg, Germany), Desmoplakin pAb (1:1000 dilution, Santa Cruz, Dallas, TX, USA), GAPDH mAb (GA1R, 1:1000 dilution, AviaSysBio, San Diego, CA, US), E-Cad mAb (36, 1:4000 dilution, BD, Heidelberg, Germany) α-Tubulin mAb (DM1A, 1:3000 dilution, Abcam, Cambridge, UK), cytokeratin 14 mAb (LL002, 1:5000 dilution, Abcam, Cambridge, UK) and HRP-coupled goat anti-rabbit or goat anti-mouse secondary antibodies (Dianova, Hamburg, Germany) were used.
Immunofluorescence was performed with Dsg1 mAb (p124, 1:100 dilution, Progen, Heidelberg, Germany), Dsg2 mAb (10G11, 1:20 dilution, Acris/OriGene Technologies, Herford, Germany), Dsg3 mAb (5G11, 1:100 dilution, Invitrogen/ThermoFisher scientific, Waltham, USA), Dsg3 pAb (1:100 dilution, Biozol, Eching, Germany), Dsc1 pAb (1:100 dilution, Abbexa, Cambridge, UK), Dsc3 mAb (U114, 1:50 dilution, Progen, Heidelberg, Germany) and Cy2- or Cy3 coupled goat anti-rabbit or goat anti-mouse secondary antibodies (Dianova, Hamburg, Germany).

Cell lysis and cell fractionation

For whole cell lysates confluent cells were washed with cold PBS, lysed in SDS-lysis buffer (25 mM HEPES, 2 mMol EDTA, 25 mM NaF and 1 % sodiumdodecylsulfate, pH 7.4) and sonicated. As desmosomes are known to be insoluble in Triton X-100, the desmosome containing fraction was isolated using Triton Assay as described before (1). Briefly confluent cells were washed with cold PBS and lysed in Triton X-lysis buffer (0.5 % Triton X-100, 50 mM MES, 25 mM EGTA, 5 mM $MgCl_2$, protease inhibitors) for 20 min on ice. Triton soluble, non-cytoskeletal fraction (supernatant) was separated from Triton insoluble, cytoskeleton/desmosome containing fraction (pellet) by centrifugation at 13,000 rpm for 10 min at 4 °C. Pellet was solved in SDS-lysis buffer. Protein concentrations were adapted using a BCA protein assay kit (Pierce/ThermoFisher Scientific, Waltham, USA) according to the manufacturer´s

protocol. Samples were analyzed by SDS-PAGE (SDS-polyacrylamide gel electrophoresis) followed by Western blot analyses as shown previously (1).

Immunoprecipitation

Co-immunoprecipitation of Dsg3 was performed with HaCaT lysates according to an adapted protocol of Hartlieb et al. (2). Briefly, confluent cells were washed and lysed in RIPA buffer (50 mM Tris-HCl, pH 8; 150 mM NaCl; 0,1 % SDS; 1 % NP-40; 1 mM EDTA and cOmplete protease inhibitors (Roche/Sigma-Aldrich, Taufkirchen, Germany)) for 15 min on ice. After centrifugation (5 min, 13,000 rpm) protein concentration of the supernatant was determined using a BCA protein assay kit (Pierce/ThermoFisher Scientific, Waltham, USA) according to the manufacturer´s protocol. Preclearing of the lysates with 30 µl washed protein A/G beads (Santa Cruz Biotechnology, Dallas, TX, USA) was done to avoid unspecific binding to the beads. IP was performed with 1 mg of precleared lysates and 1,5 µg of anti-Dsg3 pAb (Biozol, Eching, Germany), anti-Dsg2 pAb (Abbexa, Cambridge, UK) or a polyclonal rabbit ctr-IgG for 3 h at 4 °C on a rotating incubator. To precipitate antibody bound proteins, 30 µl of washed protein A/G beads were added for an overnight incubation at 4 °C and subsequent centrifugation. Beads were washed with RIPA buffer and precipitated proteins were solved in 3x Laemmli (3), boiled for 5 min at 95 °C and analyzed by Western blotting. For co-immunoprecipitation after crosslinking of surface proteins, cells were treated with the membrane impermeable, cleavable crosslinker DTSSP (3,3´-dithiobis[sulfosuccinimidylpropionate], ThermoScientific, Waltham, USA) according to the manufacturer´s instructions. Briefly, cells were washed twice with PBS and incubated with 2 mM DTSSP in PBS for 30 min at RT. Crosslinking was stopped by quenching with 20 mM Tris, pH 7.5 for 15 min and cells were washed, lysed and immunoprecipitation was performed as described above. Crosslinks were cleaved by adding Laemmli with 100 mM DTT and boiling for 5 min at 95 °C before loading on the gel.

Cell surface biotinylation

For precipitation of biotinylated cell surface proteins a protocol modified from Vielmuth et al. (4) was performed. In short, confluent cells were washed with cold PBS and incubated with membrane impermeable 0.25 mM EZ-Link Sulfo-NHS-Biotin (ThermoScientific, Waltham, USA) for 1 h on ice. After washing in PBS containing 100 mM Glycin and PBS only cells were lysed in RIPA buffer (50 mM Tris-HCl, pH 8;

150 mM NaCl; 0.1 % SDS; 1 % NP-40; 1 mM EDTA and cOmplete protease inhibitors, Roche/Sigma-Aldrich, Taufkirchen, Germany)) and centrifuged. Biotinylated surface proteins from pellet and supernatant were precipitated with NeutrAvidin HighCapacity agarose beads (ThermoScientific, Waltham, USA) under rotation over night at 4 °C. Beads were washed in lysis buffer and suspended in 3x Laemmli buffer (3) and precipitated proteins were analyzed by Western blot.

Immunofluorescence

Tissue samples from *ex vivo*- and patient skin were embedded in TissueTec (Leica Biosystems, Nussloch, Germany), subsequently frozen at -20 °C and serially sectioned at 7 µm thickness using a cryostat microtome (HM 500 OM, Microm International GmbH, Walldorf, Germany). For immunostaining, slices were heated for 30 min at 60 °C and fixed with PBS and 2 % paraformaldehyde, permeabilized with 1 % Triton X-100 for 1 h. After blocking with 3 % bovine serum albumin (VWR International, Darmstadt, Germany) and 1 % normal goat serum (Jackson ImmunoResearch, Cambridgeshire, UK) for 60 min, primary antibodies were incubated overnight at 4°C. Incubation with secondary antibodies was done for 1 h and DAPI (Roche, Penzberg, Germany) staining for 10 min at room temperature. Finally, slides were mounted with 1.5 % n-propyl gallate in glycerol and images were taken using a Leica SP5 confocal microscope with a 63x NA 1.4 PL APO objective controlled by LAS AF software (Leica, Mannheim, Germany). Secondary antibody controls were stained using Cy2- or Cy3-coupled goat anti-rabbit or goat anti-mouse antibodies only.

Patient samples and purification of IgG fractions and antibodies (Abs)

Serum and samples of patients were provided by Matthias Goebeler (Department for Dermatology, Venereology and Allergology, University hospital Würzburg), Miklós Sárdy (Department of Dermatology, Derma-oncology and Venerology, University Budapest), Michael Hertl (Department of Dermatology and Allergology, Philipps University, Marburg), and Enno Schmidt (Department of Dermatology, University of Lübeck). Sera and skin biopsies were used with informed and written consent and under approval of the local ethic committee. All patients had an active disease at the time of collection including lesions of the skin and the mucous membranes. ELISA for Dsg1 and Dsg3 were done according to the manufacturer´s protocol (Euroimmun, Lübeck, Germany (patient 2); MBL, Nagoya, Japan (all other patients). ELISA scores are given in Table 1. Cut-offs were at 20 U/ml.

IgG fractions of patients (PV-IgG) and healthy volunteers (ctr-IgG) were purified by Protein A Agarose affinity chromatography (Life technologies/ThermoFisher scientific, Waltham, USA) as described before (5). Purification of the pathogenic Dsg3 mAb, AK23 was performed from supernatants from hybridoma cells using Protein G Agarose (Millipore/Merck, Darmstadt, Germany). In brief before supernatant collection, cells were grown without FCS (RPMI 1640, 1 % L-Glutamin, 50 units/ml penicillin (AppliChem, Darmstadt, Germany), 50 µg/ml streptomycin (AppliChem)). Cell suspension was centrifuged for 15 min at 5000 rpm at 4 °C and supernatant was added to a Protein G loaded column (MoBiTec GmbH, Goettingen, Germany) to bind murine IgGs. After elution with citric acid and subsequent neutralization antibodies were concentrated using Amicon Ultra-4 centrifugal filters (Merck, Darmstadt, Germany) and antibody concentration was determined by BCA protein assay kit (Pierce/ThermoFisher Scientific, Waltham, USA) according to the manufacturer´s protocol. 75 µg/ml of AK23 were used in experiments.

Purification of recombinant Dsg-Fc constructs

Purification of recombinant Dsg-Fc proteins was carried out as described before (5). Briefly, Dsg2- or Dsg3- extracellular domain-Fc constructs were expressed stably in Chinese hamster ovarian cells (CHO cells). After reaching 65-75 % confluence, supernatant was collected and recombinant proteins were isolated by protein A agarose affinity chromatography (Life Technologies/ThermoFisher scientific, Waltham, USA; details please refer purification of Antibodies). Coomassie staining and Western blot analysis with Dsg2 mAb (10G11, Acris/OriGene Technologies, Herford, Germany) and Dsg3 mAb (clone5G11; Life Technologies) were performed to test purity.

Atomic force microscopy

AFM measurements were done using a NanoWizard 3 AFM (JPK Instruments, Berlin, Germany) mounted on an inverted optical microscope (Carl Zeiss, Jena, Germany) or a Nanowizard 4 AFM (JPK Instruments, Berlin, Germany) mounted on an inverted optical microscope (IX73 Olympus, Hamburg, Germany) with a 10x objective in a cell free setup described in detail before (6). Briefly, cantilever of Silicon Nitrid MLCT AFM probes (Bruker, Mannheim, Germany) and silicon nitride mica sheets (SPI Supplies, West Chester, USA) were functionalized with corresponding Dsg-Fc constructs as illustrated before (7). For this a flexible heterobifunctional benzaldehyde polyethylenglycol (PEG) linker (BroadPharm, San Diego, US) was used to link proteins

(0.15 mg/ml) to the tip or mica sheet respectively. Measurements were done with the triangular D-tip with nominal spring constant of 0.03 N/m and tip radius of 20 nm on a 2.5 mm^2 area with 10x10 pixel grid and a relative setpoint of 0.5 nN, a Z-length of 0.3 µm and a pulling speed of 1 µm/s. By evaluation of the resulting force-distance curves adhesive properties of specific molecules can be analyzed (8). For determination of lifetimes pulling speeds from 1 µm/s to 20 µm/s were used and extreme fitted values of UFs were blotted against loading rates and fitted in a modified bells equation (9, 10)

PCR

Genotyping of pups used for immortalization of keratinocytes was done from tail DNA. The following protocol was used: initial denaturation at 95 °C for 3 min, followed by 30 cycles of: 95 °C for 30 sec, 53 °C for 30 sec, 72 °C for 25 sec and final elongation at 72 °C for 6 min. Following 5'-3' primer sequences were used: CAAGCGTTTCTGGTGAGACC as common, TTGTTGCTGGTGTGTCTGGT as wt reverse and GCCTGAAGAACGAGATCAGC as ko reverse. PCR products were analyzed by agarose gel electrophoresis.

Supplemental references

1. E. Hartlieb, B. Kempf, M. Partilla, B. Vigh, V. Spindler and J. Waschke: Desmoglein 2 is less important than desmoglein 3 for keratinocyte cohesion. *PLoS One*, 8(1), e53739 (2013) doi:10.1371/journal.pone.0053739
2. E. Hartlieb, V. Rotzer, M. Radeva, V. Spindler and J. Waschke: Desmoglein 2 compensates for desmoglein 3 but does not control cell adhesion via regulation of p38 mitogen-activated protein kinase in keratinocytes. *J Biol Chem*, 289(24), 17043-53 (2014) doi:10.1074/jbc.M113.489336
3. U. K. Laemmli: Cleavage of structural proteins during the assembly of the head of bacteriophage T4. *Nature*, 227(5259), 680-5 (1970) doi:10.1038/227680a0
4. F. Vielmuth, J. Waschke and V. Spindler: Loss of Desmoglein Binding Is Not Sufficient for Keratinocyte Dissociation in Pemphigus. *J Invest Dermatol*, 135(12), 3068-3077 (2015) doi:10.1038/jid.2015.324
5. W. M. Heupel, D. Zillikens, D. Drenckhahn and J. Waschke: Pemphigus vulgaris IgG directly inhibit desmoglein 3-mediated transinteraction. *J Immunol*, 181(3), 1825-34 (2008) doi:10.4049/jimmunol.181.3.1825
6. F. Vielmuth, M. T. Wanuske, M. Y. Radeva, M. Hiermaier, D. Kugelmann, E. Walter, et al.: Keratins Regulate the Adhesive Properties of Desmosomal Cadherins through Signaling. *J Invest Dermatol*, 138(1), 121-131 (2018) doi:10.1016/j.jid.2017.08.033

7. A. Ebner, L. Wildling, A. S. Kamruzzahan, C. Rankl, J. Wruss, C. D. Hahn, et al.: A new, simple method for linking of antibodies to atomic force microscopy tips. *Bioconjug Chem*, 18(4), 1176-84 (2007) doi:10.1021/bc070030s
8. F. A. Carvalho and N. C. Santos: Atomic force microscopy-based force spectroscopy--biological and biomedical applications. *IUBMB Life*, 64(6), 465-72 (2012) doi:10.1002/iub.1037
9. G. Bell: Models for the specific adhesion of cells to cells. *Science*, 200(4342), 618-627 (1978) doi:10.1126/science.347575
10. J. A. Wieland, A. A. Gewirth and D. E. Leckband: Single Molecule Adhesion Measurements Reveal Two Homophilic Neural Cell Adhesion Molecule Bonds with Mechanically Distinct Properties. *Journal of Biological Chemistry*, 280(49), 41037-41046 (2005) doi:10.1074/jbc.M503975200

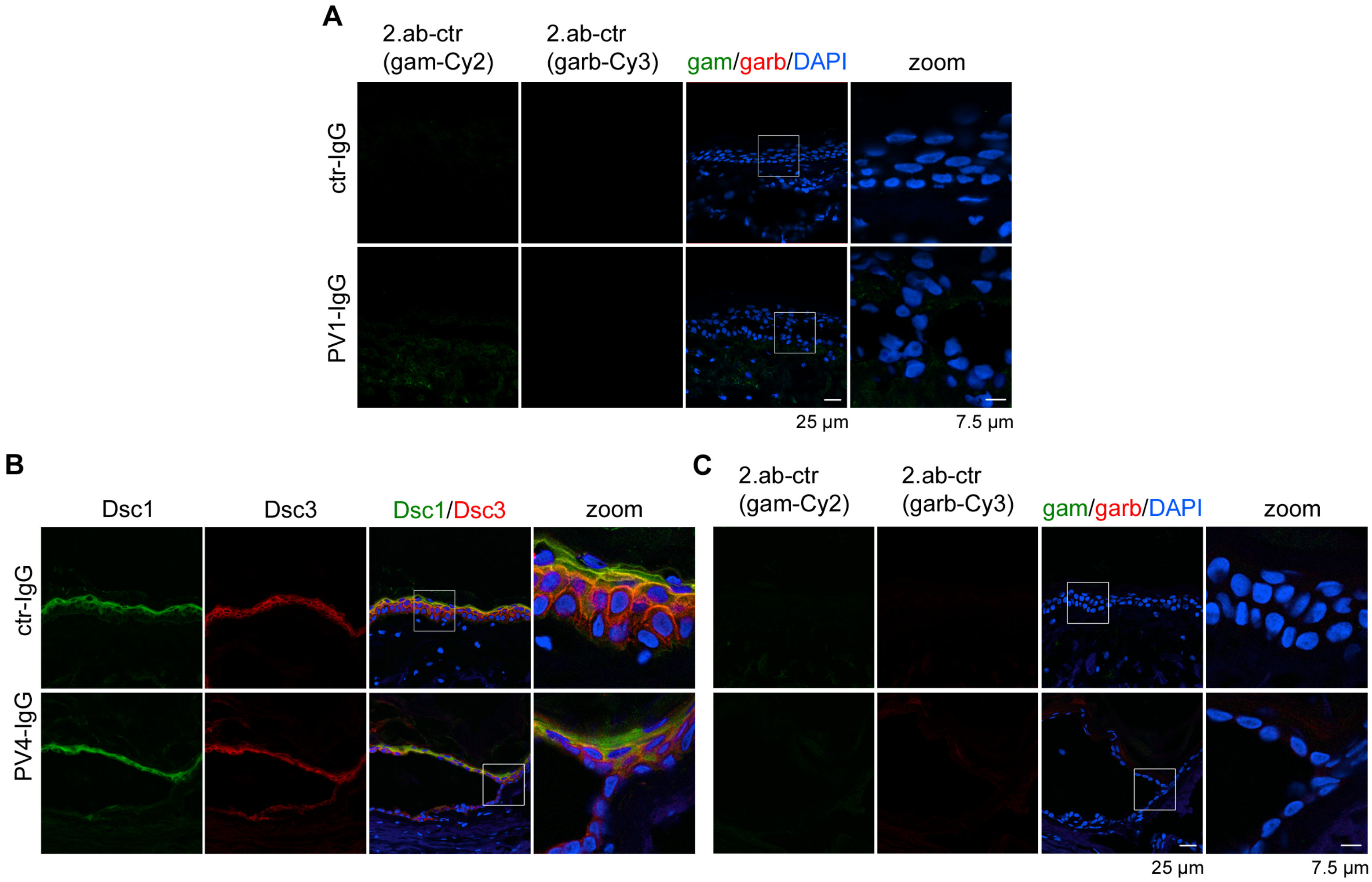

Fig S1: Secondary antibody controls and Dsc staining in *ex vivo* pemphigus model
(A) Immunostaining with secondary antibodies only in human *ex vivo* skin after injection with PV1-IgG or control (ctr) IgG from healthy humans (n=3, with PV1-IgG and PV2-IgG) confirming specificity of used primary antibodies. Representative immunostaining against Dsc1 and Dsc3 **(B)** and secondary antibodies only **(C)** in human *ex vivo* skin after injection with PV4-IgG or ctr-IgG from healthy humans (n=3, with PV1-, PV2- and PV4-IgG) showing no elevation of Dsc1 and Dsc3 staining. Nuclei were stained with DAPI. desmocollin (Dsc), pemphigus vulgaris (PV), secondary antibody control (2.ab-ctr), goat anti-mouse (gam), goat anti-rabbit (garb).

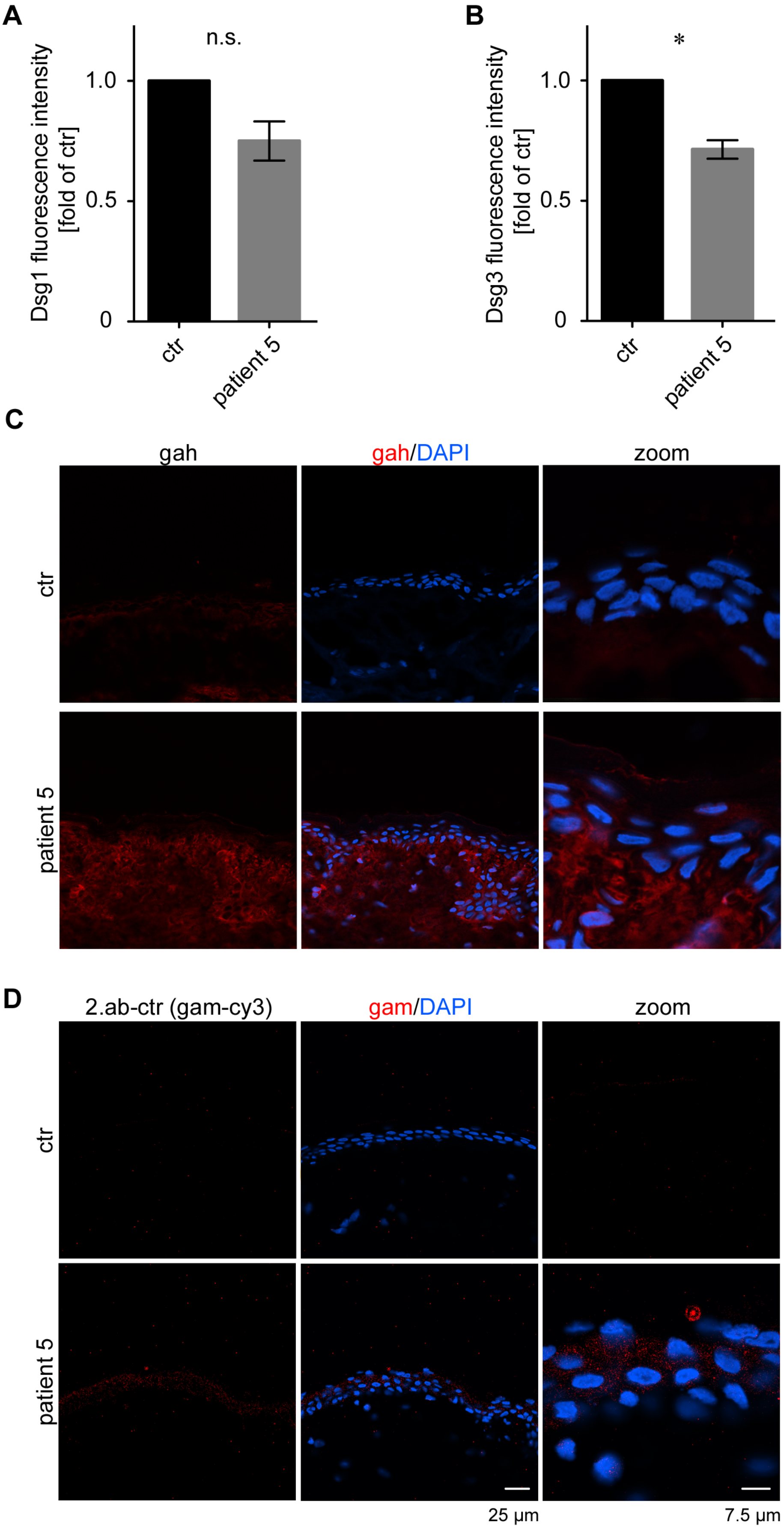

Fig S2: Quantifications and secondary antibody controls
Quantification of Dsg1 **(A)** and Dsg3 **(B)** staining of patient 5 showing mild depletion of the respective proteins. Samples were stained against human IgG **(C)** and secondary antibody only **(D)** to confirm autoantibody binding and specificity of used primary antibodies respectively. Untreated *ex vivo* skin served as a control. Nuclei were stained with DAPI. Columns indicate mean value normalized to ctr ± SEM, *P<0.05; student t-test to ctr. desmocollin (Dsc), pemphigus vulgaris (PV), secondary antibody control (2.ab-ctr), goat anti-human (gah), goat anti-mouse (gam), goat anti-rabbit (garb).

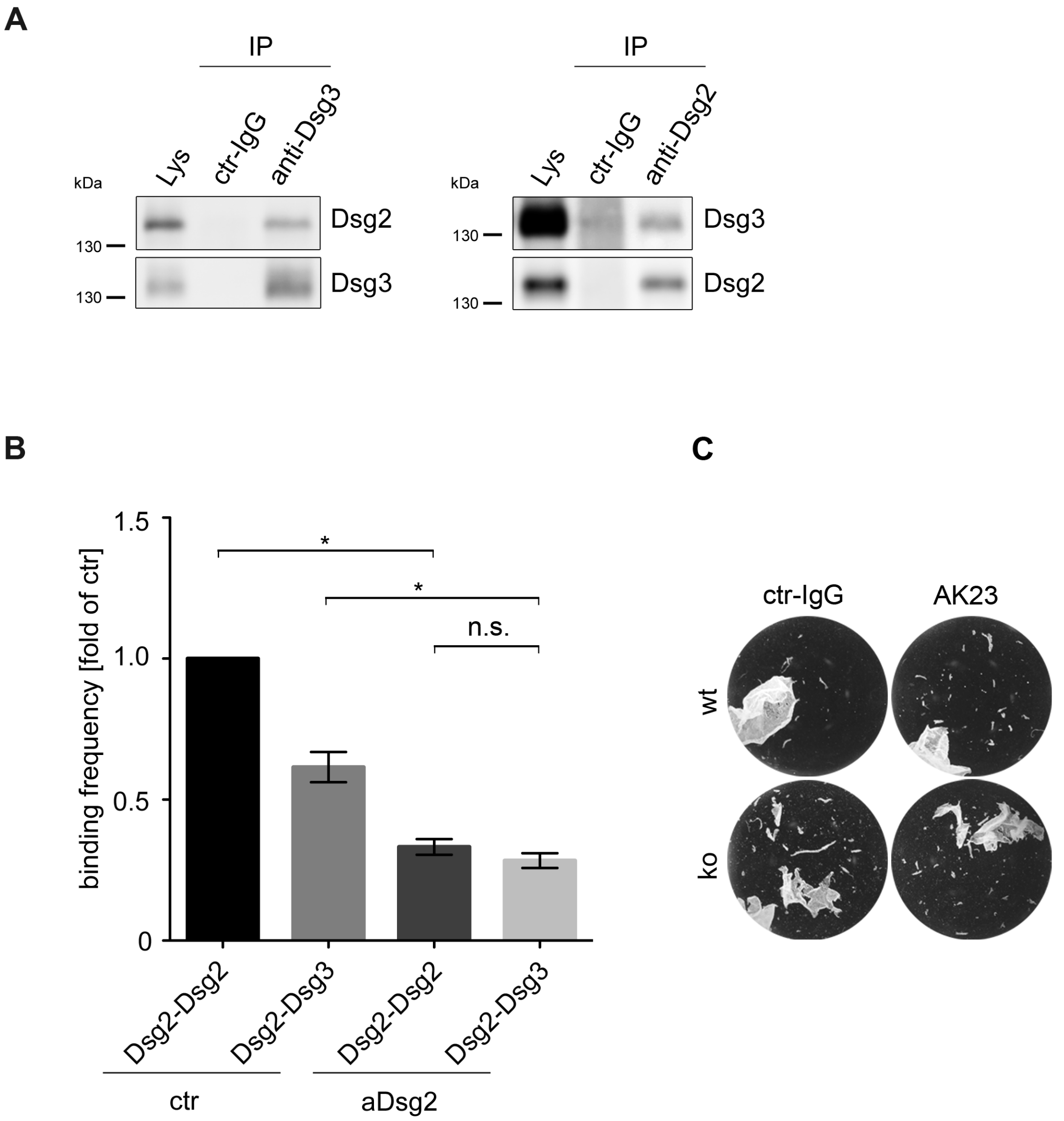

Fig S3: Co-immunoprecipitation, AFM measurements and Dispase-based keratinocyte assay after aDsg2 antibody incubation.

(A) IP in human keratinocytes (HaCaT) with a polyclonal anti-Dsg3 or anti-Dsg2 antibody or rabbit control (ctr) IgG after surface crosslinking with the membrane impermeable crosslinker DTSSP showing heterophilic interactions of Dsg2 and Dsg3. Pure lysates (Lys) were loaded as a control. Representative of n=3 **(B)** Binding frequencies of cell-free AFM measurements probing Dsg2-Dsg2 and Dsg2-Dsg3 interaction pairs with and without (ctr) incubation of an inhibitory anti-Dsg2 antibody (aDsg2) for 1 h. (n=5 tip/sample combinations (500 force distance curves) each from 3 independent coating procedures). aDsg2 blocked both homo- and heterophilic interactions.
(C) Photographs of dispase-based keratinocyte dissociation assay of Dsg3 wt and ko keratinocytes after treatment with control (ctr) IgG or a monoclonal anti-Dsg3 antibody derived from a pemphigus mouse model (AK23) for 24 h (n=3). Columns indicate mean value normalized to ctr ± SEM, *P<0.05; One-way ANOVA with Bonferroni correction. desmoglein (Dsg)
